# A Polymer Electrolyte with Rigid–Flexible Coupled Architecture for High-Voltage Lithium-Metal Batteries

**DOI:** 10.3390/polym18080987

**Published:** 2026-04-18

**Authors:** Haoru Xie, Zhengyin Yao, Zhen Liu, Ruiyong Chen, Peng Zhang

**Affiliations:** 1 Key Laboratory for Polymeric Composite and Functional Materials of Ministry of Education, School of Materials Science and Engineering, Institute of Green Chemistry and Molecular Engineering, Sun Yat-sen University, Guangzhou 510275, China; 2 Medical Devices Research & Testing Center, South China University of Technology, Guangzhou 510006, China; 3 Department of Chemistry, University of Liverpool, Liverpool L7 3NY, UK

**Keywords:** polymer electrolyte, lithium metal battery, solution casting, in situ polymerization, eutectic electrolyte

## Abstract

A polymer electrolyte is developed by integrating a poly(methyl methacrylate) (PMMA)/eutectic electrolyte (EE) phase into a porous polyethylene (PE) scaffold via a solution-casting strategy. In this rigid–flexible coupled architecture, the PMMA matrix serves as a solid host that coordinates with Li^+^ through its polar carbonyl groups, thereby promoting lithium salt dissociation and establishing a stable ion transport network. The incorporated EE, composed of ethylene carbonate and LiTFSI, effectively reduces the glassy rigidity of PMMA and provides continuous pathways for fast ionic conduction. Meanwhile, the porous PE scaffold reinforces mechanical strength and resists lithium dendrite penetration, enabling a thin electrolyte membrane with excellent flexibility. The resulting electrolyte achieves an ionic conductivity of 1.59 × 10^−4^ S cm^−1^ at 30 °C, a lithium-ion transference number of 0.45, and an electrochemical stability window up to 4.75 V. In Li||LiFePO_4_ cells, it delivers stable cycling at 3 C for 1000 cycles with 76.8% capacity retention and a Coulombic efficiency exceeding 99.9%. The monomer-free design eliminates residual reactive species that commonly compromise interfacial stability, offering a reliable pathway toward high-voltage solid-state lithium-metal batteries.

## 1. Introduction

The electric vehicle industry has rapidly emerged in recent years, bringing with it an urgent demand for efficient energy storage devices [1,2,3]. Lithium-ion batteries have garnered widespread attention due to their high long-term cycling stability and high energy density [4,5]. The core of research into achieving novel high-efficiency energy storage lithium-metal batteries lies in the development of solid-state electrolytes that possess high energy density, high safety, and good compatibility with electrodes [6]. Current research on electrolytes primarily focuses on two aspects: inorganic solid-state electrolytes and polymer solid-state electrolytes [7,8]. Inorganic solid-state electrolytes exhibit relatively superior mechanical strength and ionic conductivity. However, their high rigidity leads to poor interfacial contact between the inorganic solid electrolytes and the electrodes, which impedes ionic conduction within the battery [9]. In contrast, polymer electrolytes can form relatively intimate contact with electrodes, yet they suffer from significant drawbacks such as an inability to suppress lithium dendrite growth and relatively low ionic conductivity [10,11].

Eutectic electrolytes (EEs) have attracted significant attention due to their low volatility, enhanced safety, and favorable cycling stability [12,13,14]. Among them, the EE system composed of ethylene carbonate (EC) and lithium bis(trifluoromethanesulfonyl)imide (LiTFSI) exhibits low volatility, and EC itself is non-flammable, offering safety advantages over conventional carbonate-based electrolytes [15]. However, EE systems typically suffer from high viscosity, resulting in lower ionic conductivity compared to conventional carbonate electrolytes, which limits their further application [16]. On the other hand, poly(methyl methacrylate) (PMMA), owing to its polar carbonyl groups, can coordinate with Li^+^ and promote lithium salt dissociation [17,18]. It is also low-cost and easy to process, making it commonly used in polymer electrolyte research [19]; nevertheless, the glassy nature of PMMA significantly suppresses room-temperature ionic transport [20]. Furthermore, when PMMA absorbs liquid to form gel polymer electrolytes, the mechanical strength often decreases, potentially compromising dendrite penetration resistance and safety [21]. Recent studies on elastomer-based polymer electrolytes suggest that introducing liquid or quasi-liquid components can enhance ionic conductivity [22], but this usually leads to new trade-offs, such as deteriorated mechanical properties or aggravated interfacial side reactions [23]. Therefore, achieving a synergistic balance among ionic conductivity, mechanical toughness, and dendrite resistance remains a core challenge in the design of polymer electrolytes.

A polymer electrolyte was designed and fabricated, composed of a PMMA polymer matrix, EC/LiTFSI EE, and a rigid porous polyethylene (PE) support. In this design, the PMMA matrix ensures the solidification of the flexible components and hosts the EE, working synergistically to achieve complete dissociation of the lithium salt and construct a complete and stable ion transport network [19,24]. The EE ensures favorable ionic conductivity of the electrolyte. The PE skeleton effectively enhances the mechanical properties of the electrolyte and strengthens its resistance to lithium dendrite penetration, enabling stable operation even with a relatively small thickness. Ultimately, a polymer electrolyte with an ionic conductivity of 1.59 × 10^−4^ S cm^−1^ at room temperature was obtained. Batteries assembled with a LiFePO_4_ cathode delivered a capacity of 102 mAh g^−1^ at a 3 C rate, with a capacity retention of 76.8% after 1000 cycles and a Coulombic efficiency exceeding 99.9%. Meanwhile, during the experimental exploration, it was found that compared with in situ polymerization, the polymer electrolyte prepared by the solvent casting method seemed to have better performance, and certain experiments were designed to explore this phenomenon.

## 2. Materials and Methods

### 2.1. Materials

The following chemicals were used as received: methyl methacrylate (MMA, purity 99.0%, Shanghai Macklin Biochemical Technology Co., Ltd., Shanghai, China), poly(ethylene glycol) ethylene carbonate (EC, purity 99.0%, Shanghai Aladdin Biochemical Technology Co., Ltd., Shanghai, China), N-Methyl-2-pyrrolidone (NMP, electronic grade, purity 99.9%, Shanghai Aladdin Biochemical Technology Co., Ltd., Shanghai, China), lithium bis(trifluoromethanesulfonyl)imide (LiTFSI, purity 99.0%, Shanghai Aladdin Biochemical Technology Co., Ltd., Shanghai, China), poly(ethylene glycol) diacrylate (PEGDA, Mn = 700, purity 99.0%, Shanghai Macklin Biochemical Technology Co., Ltd., Shanghai, China), and 2,2′-Azobis(2-methylpropionitrile) (AIBN, purity 99.0%, Shanghai Aladdin Biochemical Technology Co., Ltd., Shanghai, China). Additional materials, including LiFePO_4_ (LFP, electronic grade), LiCoO_2_ (LCO, electronic grade), poly(vinylidene fluoride) (PVDF, Mw = 455,000 Da), Super P carbon black (electronic grade), polypropylene diaphragms (electronic grade), lithium metal foil, stainless steel (SS) plate, aluminum (Al) foil, and 2025-type coin cell components (case, gasket, spacer, wave spring, and cap) were purchased from Guangdong Canrd New Energy Technology Co., Ltd., Dongguan, China.

### 2.2. Preparation of PMMA-Based Electrolyte Membranes

The polymer electrolyte membrane was prepared using a dip-coating method. Briefly, PMMA was dissolved in THF and stirred at 30 °C for 24 h to form a homogeneous solution. Subsequently, the EE, formed by EC and LiTFSI, was added according to a fixed mass ratio. The samples were labeled as PLE_1x_, where the mass ratio of LiTFSI:EC was 1:2.4, and the mass ratio of PMMA:EE was 1:x. The PE separator was immersed in the solution to form a wet electrolyte membrane, which was then dried for 24 h to obtain the final polymer electrolyte membrane. To optimize the electrolyte performance, a series of compositions were investigated, and the PLE_12_ sample (mass ratio PMMA:LiTFSI:EC = 250:147:353) exhibited the most superior comprehensive performance (Supporting Information Figures S1 and S2).

The polymer electrolyte membrane was also prepared using an in situ synthesis method. Briefly, MMA, LiTFSI, and EC in a mass ratio of 250:147:353 were mixed and stirred at 30 °C for 24 h to form a homogeneous solution. Subsequently, 0.5 wt.% AIBN (relative to the fixed weight of MMA in the sample) was added as an initiator, and 0.5 wt.% PEGDA (relative to the fixed weight of MMA in the sample) was added as a crosslinking agent. After homogenization by stirring, the PE separator was immersed in the solution to form a wet electrolyte membrane, which was then heated at 70 °C for 12 h to obtain the final polymer electrolyte membrane, which was labeled as in situ PLE_12_, All operations were conducted in an argon-filled inert atmosphere glove box (O_2_ and H_2_O contents < 0.01 ppm).

### 2.3. Preparation of Cathode Materials

LFP or LCO and NCM811 cathodes based on PVDF were prepared. Briefly, 80 wt.% LFP or LCO, 10 wt.% Super P, and 10 wt.% PVDF were mixed in NMP to form a homogeneous slurry. The slurry was coated onto aluminum foil and dried in a vacuum oven at 80 °C for 24 h. The aluminum foil was then punched into cathodes with a diameter of 12 mm, and the active material loading was approximately 1.2 mg·cm^−2^.

### 2.4. Electrochemical Measurements

All electrochemical measurements were carried out using a CHI660 electrochemical workstation (Shanghai Chenhua Instrument Co., Ltd., Shanghai, China). The ionic conductivity of the electrolyte membranes was determined by electrochemical impedance spectroscopy (EIS) over a frequency range from 0.1 Hz to 10 MHz with an applied alternating current (AC) amplitude of 10 mV.

For the conductivity measurements, symmetric cells were assembled using two polished stainless steel blocking electrodes (SS|electrolyte|SS) inside an argon-filled glove box. Prior to testing, all cells were equilibrated at room temperature for 12 h to ensure stable interfacial contact. The ionic conductivity (*σ*) was calculated according to Equation (1).

*σ* = *d*/*RA*, (1)

where *d* (cm) is the thickness of the polymer electrolyte membrane, *A* (cm^2^) is the effective electrode area, and *R* (Ω) is the bulk resistance obtained from EIS measurements. (The thickness of the electrolyte membrane is 39 μm, and the diameter of the stainless steel electrode is 16 mm.) The temperature dependence of the ionic conductivity was analyzed using the Arrhenius equation (Equation (2)):

*σ* = *Aexp* (−*E_a_*/*RT*), (2)

where *σ* is the ionic conductivity, *E_a_* is the activation energy, *A* is the pre-exponential factor, *R* is the universal gas constant, and *T* is the absolute temperature.

The electrochemical stability window of the solid-state electrolyte was evaluated at room temperature by linear sweep voltammetry (LSV) using stainless steel as the working electrode and lithium metal as the counter electrode. The measurements were conducted at a scan rate of 1 mV·s^−1^ over a voltage range of 2.0–6.0 V. The lithium-ion transference number (t_(_Li^+^_)_) was determined using the chronoamperometric method in Li|electrolyte|Li symmetric cells under an applied potential of S10 mV. The value of t_(_Li^+^_)_ was calculated according to the following Equation (3):

*t*_*Li*+_ = *I_s_*(Δ*V* − *I*_0_*R*_0_)/*I*_0_((Δ*V* − *I_s_R_s_*), (3)

where Δ*V* (V) is the applied potential, *I*_0_ and *I_s_* are the initial and steady-state currents, respectively, and *R*_0_ and *R_s_* represent the initial and steady-state interfacial resistances of the passivation layer, as obtained from EIS measurements.

### 2.5. Battery Assembly and Electrochemical Measurements

Coin-type cells (CR2025) were assembled in an argon-filled inert atmosphere glove box (O_2_ < 0.1 ppm, H_2_O < 0.1 ppm). All electrochemical measurements were performed using CR2025 coin cells. Galvanostatic charge/discharge tests were conducted using a LAND-CT3002A battery tester (Wuhan LAND Electronic Co., Ltd., Wuhan, China). The voltage ranges were 2.4–4.0 V for LFP cathodes, 3.0–4.5 V for LCO cathodes, and 3.0–4.5 V for NCM811 cathodes. The 1C rate was defined as 170 mAh·g^−1^ for LFP and 200 mAh·g^−1^ for LCO/NCM811.

### 2.6. Material Characterization

#### 2.6.1. Mechanical Stress–Strain Test

Tensile stress tests were performed using a WDW-5A testing machine (Panasonic, Osaka, Japan). The membrane samples were cut into a dumbbell shape (width: 2 mm, length: approximately 14 mm), and the tensile rate was 10 mm min^−1^.

#### 2.6.2. Electrolyte Membrane Thickness Measurement

The thickness of the electrolyte membrane was measured using an AWT-CHY01 thickness gauge (EVERTE Co., Ltd., Dongguan, China).

#### 2.6.3. Thermogravimetric Analysis

The prepared samples were heated at 120 °C (above the boiling point of MMA, which is approximately 100 °C) for 12 h. The mass difference before and after heating was measured for both the in situ polymerized samples and the solvent-cast samples. The mass difference in the solvent-cast samples before and after heating was subtracted from that of the in situ polymerized samples to obtain the remaining MMA mass in the in situ polymerized samples.

#### 2.6.4. Raman Spectroscopy

Raman data of the samples were collected using a Zolix Finder930 Raman spectrometer (Zolix Instruments Co., Ltd., Beijing, China) with a laser wavelength of 532 nm.

#### 2.6.5. Fourier Transform Infrared Spectroscopy

Infrared spectra of the samples were tested using an IS50 Micro-IR micro-infrared spectrometer (Thermo Fisher Scientific, Waltham, MA, USA). The thin film samples were directly tested using the attenuated total reflectance (ATR) method. Powder samples were tested after mixing uniformly with KBr and pressing into pellets.

#### 2.6.6. Scanning Electron Microscopy

The microscopic morphology of the samples was observed using a Carl Zeiss instrument (Carl Zeiss AG, Oberkochen, Germany) operated at 50 kV. The samples were sputter-coated with platinum prior to testing. Sample test conditions: acceleration voltage was set to 10 kV.

### 2.7. Software

SetupLAND_7.4.7.0 for LAND-CT3002A battery tester.

chi660e 22.4.0.0 for CHI660 electrochemical workstation.

## 3. Results and Discussion

### 3.1. Mechanical and Electrochemical Properties

**Figure 1:**
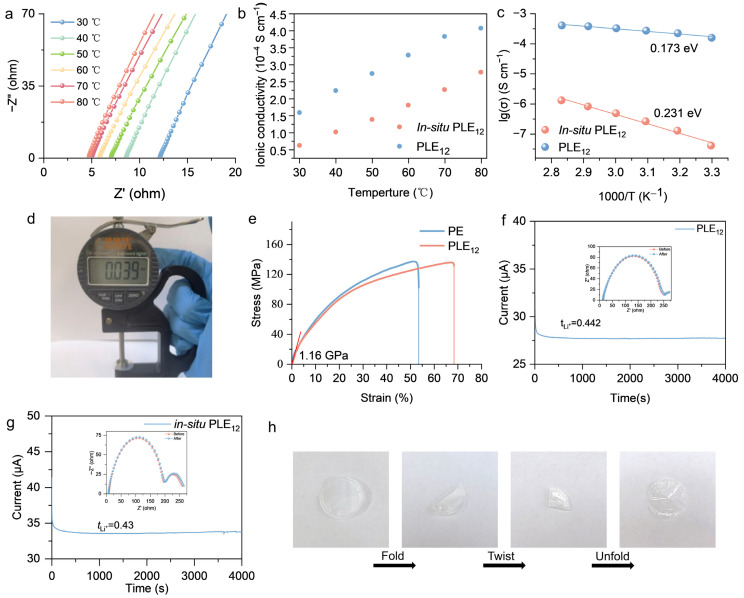
(**a**) Electrochemical impedance spectroscopy curves of PLE_12_ between 30 °C and 80 °C; (**b**) Ionic conductivity of PLE_12_ and in situ PLE_12_ between 30 and 80 °C; (**c**) Arrhenius plots of ionic conductivity for PLE_12_ and in situ PLE_12_ from 30 °C to 80 °C; (**d**) Optical photograph of PLE_12_ electrolyte membrane thickness; (**e**) Tensile test results of PE scaffold and PLE_12_ electrolyte membrane; (**f**) Lithium-ion transference number measurement result of PLE_12_ electrolyte membrane; (**g**) Lithium-ion transference number measurement result of in situ PLE_12_ electrolyte membrane; (**h**) Optical photograph of PLE_12_ electrolyte membrane upon repeated folding and unfolding.

Achieving a balance between mechanical properties and high ionic conductivity in polymer electrolytes is of significant importance for the advancement of lithium batteries [25]. In this work, PMMA was selected as the polymer matrix for the polymer electrolyte. As a common polymer electrolyte matrix, PMMA’s unique molecular structure endows the material with favorable ionic conductivity, excellent electrode interface stability, and a high lithium-ion transference number. However, the low glass transition temperature and high rigidity of PMMA at room temperature result in poor interfacial contact between the electrolyte and the electrodes [26]. To address these issues, an EE system was incorporated with PMMA to reduce rigidity and optimize toughness, while simultaneously enhancing the ionic conductivity of the electrolyte. A commercial PE separator was introduced as a skeleton during fabrication to strengthen the mechanical properties of the electrolyte and improve its puncture resistance. To balance the ionic conductivity, mechanical properties, and long-term cycling stability of the electrolyte, polymer electrolyte with different component ratios (PLE_1x_, where 1x denotes the mass ratio of PMMA:EE was 1:x while the mass ratio of LiTFSI:EC was set at 1:2.4), the mass ratio of EC to LiTFSI was determined by referencing a previous work were prepared [13], SS|PLE_1x_|SS symmetric cells were assembled for EIS tests. The test results indicated that the impedance of the electrolyte gradually decreased with increasing EE content (Figure S1). However, long-term cycling tests of assembled LFP|PLE_1x_|Li cells revealed a different trend. When the EE-to-PMMA ratio was below 2, increasing EE content improved the cell’s cycling performance, particularly evident in the difference in discharge specific capacity. Nevertheless, when the EE-to-PMMA ratios were higher than 2, although the polymer electrolyte with a higher EE proportion exhibited a higher initial specific capacity, its long-term cycling stability deteriorated, and the capacity retention decreased significantly compared to PLE_12_ (Figure S2). This is potentially attributable to the transition of the electrolyte from a solid state towards a liquid state as the EE ratio increases, which exacerbates the side reaction of EC with the lithium metal anode [27]. To balance the ionic conductivity and long-term cycling stability of the electrolyte, PLE_12_ was selected as the optimal formulation for subsequent testing. To evaluate the ionic conductivity of PLE_12_ at various temperatures, SS|PLE_12_|SS cells assembled with PLE_12_ were subjected to EIS testing at different temperatures. Concurrently, identical tests were performed on PLE_12_-based cells prepared via in situ polymerization for comparison. The results demonstrated that PLE_12_ prepared by the solution casting method achieved an ionic conductivity of 1.59 × 10^−4^ S cm^−1^ at 30 °C, whereas that prepared by in situ polymerization exhibited only 0.614 × 10^−4^ S cm^−1^ at the same temperature (Figure 1a,b). The activation energies of the two polymer electrolytes were calculated using the Arrhenius equation [28]. The activation energy of PLE_12_ was notably lower than that of in situ PLE_12_ (Figure 1c), which correlates with the ionic conductivity test results. During the preparation of the in situ PLE_12_ sample, it was found that many bubbles appeared in the sample after polymerization of the precursor solution. This was because the polymerization of MMA needed to be completed at 70 °C, and at this temperature, unpolymerized MMA volatilized rapidly, which might be the reason why the ionic conductivity of in situ PLE_12_ was lower than that of PLE_12_. These bubbles formed insulating voids, reducing the ionic conductivity of the electrolyte and increasing the activation energy for ion migration (Figure S3). In the traditional solvent casting method, the solvent used to dissolve the polymer ultimately needs to be completely removed. In our system, however, PMMA can actually be dissolved solely by EE, and the added THF serves to a greater extent to accelerate the dissolution rate and adjust the viscosity of the slurry, allowing the slurry to flow well and fill the polymer framework at room temperature. The main advantage of preparation by this method compared with the traditional solvent casting method is that when the electrolyte is heated to remove THF, it possesses a certain fluidity as the temperature increases. After the solvent volatilizes, this fluidity allows the electrolyte to automatically fill the tiny bubbles left by THF volatilization, making the electrolyte denser and more continuous.

By adjusting the slurry concentration to balance the ionic conductivity and thickness of the polymer electrolyte, the thickness of the as-prepared electrolyte was measured to be approximately 39 μm (Figure 1d). Notably, this thickness is thinner than that of conventional polymer electrolytes, which typically exceed 50 μm [29]. Reducing the electrolyte thickness helps shorten the Li^+^ migration path, thereby decreasing the ohmic polarization of the battery during long-term cycling, which is beneficial for achieving superior performance upon extended cycling [30]. Tensile tests were conducted on the electrolyte at this thickness and compared with a commercial PE separator. PLE_12_ exhibited no significant difference in Young’s modulus compared to the PE separator and possessed a similar stress strength. However, its maximum elongation was significantly greater than that of the PE separator, reaching 68.38% (Figure 1e). This indicates that the PLE_12_ electrolyte achieved excellent ductility by combining the PMMA and EE system, corresponding to an enhancement in the toughness of PMMA. To fully demonstrate the excellent mechanical properties of PLE_12_, a PLE_12_ electrolyte without the PE separator scaffold was prepared and subjected to tensile testing. Its length before stretching was approximately 24 mm, while the maximum length after stretching was approximately 93 mm, resulting in a strain value of 287.5% (Figure S4). The excellent tensile performance of PLE_12_ demonstrates that the strategy of adding EE to PMMA effectively achieved the purpose of softening PMMA, endowing it with outstanding stretchability. Complementing these quantitative results, the electrolyte membrane also exhibited remarkable qualitative robustness, maintaining a crack-free surface even after repeated folding (Figure 1h). This inherent flexibility, a direct consequence of the rigid–flexible coupled design, is crucial for ensuring conformal interfacial contact and withstanding mechanical stresses during battery assembly and cycling.

To provide a more comprehensive comparison of the electrolytes prepared by different methods, the lithium-ion transference numbers of PLE_12_ and in situ PLE_12_ were measured. The lithium-ion transference number of PLE_12_ was 0.44, which is higher than the 0.43 of in situ PLE_12_ (Figure 1f,g). PLE_12_ not only possesses high ionic conductivity but also exhibits a higher lithium-ion transference number (0.44) compared to in situ PLE_12_ (0.43). This indicates that, compared to in situ PLE_12_, a higher proportion of the current in PLE_12_ is contributed by lithium-ion migration. This feature is advantageous for mitigating concentration polarization, thereby enhancing the rate capability and accessible capacity of the battery [31].

### 3.2. Mechanism of LiTFSI Dissociation

**Figure 2:**
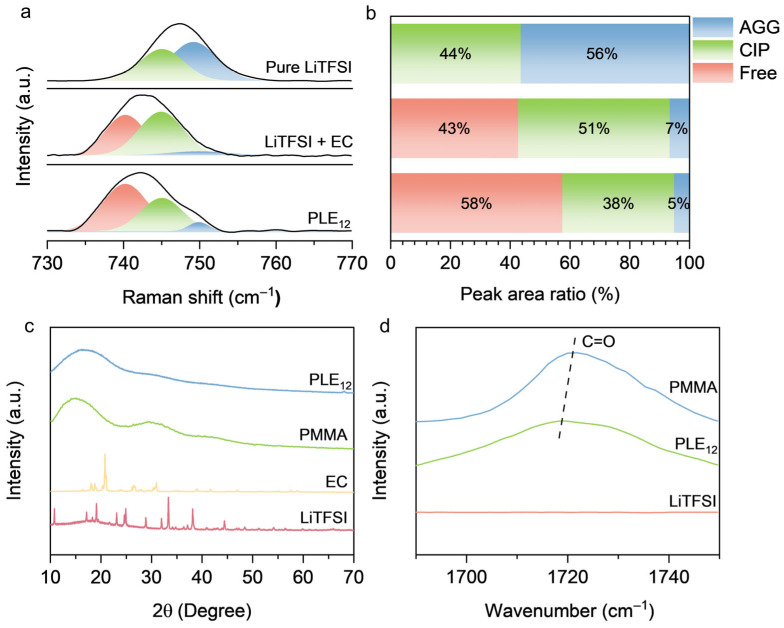
(**a**) Raman spectra and peak fitting results of pure LiTFSI, LiTFSI + EC, and PLE_12_. (**b**) Proportions of different TFSI^−^ existing states based on Raman fitting results. (**c**) X-ray diffraction patterns of PLE_12_, PMMA, EC, and LiTFSI. (**d**) Fourier transform infrared spectra of PMMA, PLE_12_, and LiTFSI.

Beyond the macroscopic mechanical and conductive properties, the synergistic interaction between the PMMA matrix and the EE phase fundamentally governs the dissociation of LiTFSI, which is critical for generating free charge carriers. To investigate the mechanism of LiTFSI dissociation within PLE_12_, Raman spectroscopic analysis was performed on pure LiTFSI, the EE composed of LiTFSI and EC, and PLE_12_. In the spectra, the peak at 740 cm^−1^ represents free TFSI^−^ anions originating from dissociated LiTFSI. The peak at 745 cm^−1^ corresponds to the contact ion pair (CIP) solvation structure, representing complete ion pairs formed between Li^+^ and TFSI^−^ with one-to-one coordination. The peak at 750 cm^−1^ represents the aggregate (AGG) solvation structure, where Li^+^ and TFSI^−^ form clusters through non-covalent interactions [32]. The proportions of these three species indicate the degree of LiTFSI dissociation (Figure 2a). Peak fitting was applied to the Raman results. Pure LiTFSI primarily consisted of AGG and CIP structures. In contrast, within the EE formed by LiTFSI and EC, significant dissociation of LiTFSI occurred, with nearly half of the TFSI^−^ existing in a free state, demonstrating that EC effectively dissociates LiTFSI. In PLE_12_, the proportion of free TFSI^−^ further exceeded half, proving that within the electrolyte, PMMA and EC synergistically achieve effective dissociation of LiTFSI (Figure 2b). The effective dissociation of LiTFSI in PLE_12_ was further corroborated by XRD analysis [33]. Sharp and prominent crystalline peaks were observed in the XRD patterns of pure LiTFSI and EC. However, no distinct crystalline peaks were present in the pattern of PLE_12_, indicating that strong hydrogen bonding between LiTFSI and EC components disrupts their respective original high-melting-point crystal lattices, forming a new hydrogen-bonded network structure with a lower melting point and achieving dissociation within the electrolyte [34]. Additionally, the XRD pattern of pure PMMA exhibited a pronounced amorphous halo, whereas the intensity of this amorphous feature was significantly weakened in PLE_12_, with no crystalline peaks emerging (Figure 2c). This suggests that the short-range ordered structure of PMMA is further disrupted in PLE_12_, increasing its disorder and consequently enhancing the ionic conductivity of PMMA. To elucidate the mechanism by which PMMA contributes to LiTFSI dissociation, Fourier transform infrared spectroscopy (FTIR) measurements were conducted on PMMA, PLE_12_, and LiTFSI [35]. The results clearly showed that pure PMMA exhibits a characteristic absorption peak at 1727 cm^−1^, which corresponds to the carbonyl group (C=O) [36] shifted to a lower wavenumber in PLE_12_ (Figure 2d). This indicates a significant interaction between the carbonyl groups in PMMA and Li^+^ in LiTFSI, which promotes the dissociation of LiTFSI within PLE_12_.

### 3.3. Compatibility with Lithium Metal

**Figure 3:**
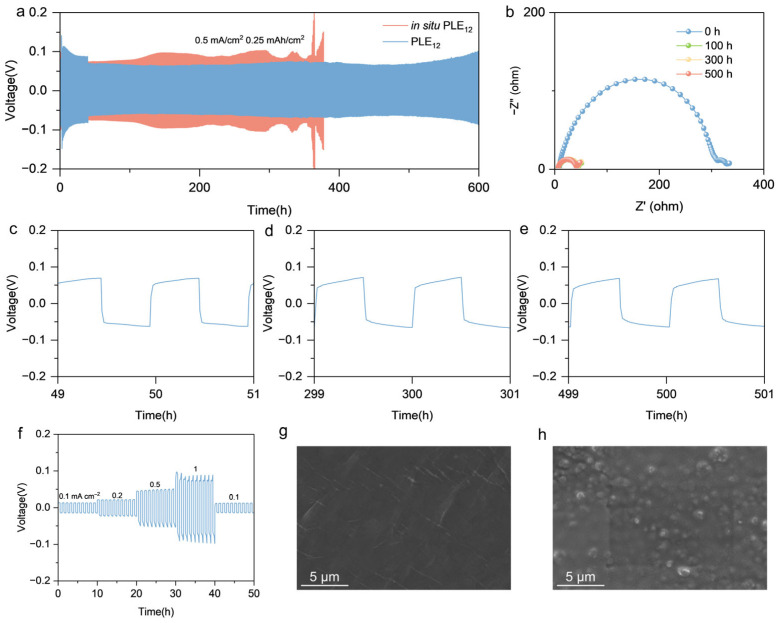
(**a**) Galvanostatic plating/stripping voltage profile of a Li|PLE_12_|Li symmetric cell cycled at a current density of 0.5 mA cm^−2^ for 600 h at 30 °C. (**b**) Electrochemical impedance spectroscopy curves of the Li|PLE_12_|Li cell after different numbers of cycles. (**c**–**e**) Enlarged voltage profiles corresponding to the regions indicated in (**a**). (**f**) Galvanostatic plating/stripping voltage profiles of a Li|PLE_12_|Li symmetric cell cycled at current densities of 0.1 mA cm^−2^, 0.2 mA cm^−2^, 0.5 mA cm^−2^, and 1 mA cm^−2^ for 10 h each. (**g**) Surface morphology of the lithium metal before cycling. (**h**) Surface morphology of the lithium metal after cycling for 400 h at a current density of 0.5 mA cm^−2^.

To verify the compatibility of PLE_12_ with lithium metal, Li|PLE_12_|Li/Li|in situ PLE_12_|Li symmetric cells were assembled for long-term cycling tests. At a current density of 0.5 mA cm^−2^ and a plating/stripping areal capacity of 0.25 mAh cm^−2^, the Li|in situ PLE_12_|Li experienced a volatile cycling process with complete failure at 400 cycles, whereas the Li|PLE_12_|Li maintained a stable polarization voltage of approximately 0.06 V throughout 600 cycles without significant fluctuations (Figure 3a). To investigate the cause of this phenomenon, a lithium sheet was brought into contact with MMA to test the reactivity between them. From the testing process, it could be observed that the surface of the lithium sheet changed from a shiny state to a rough black state after contacting MMA. Such high reactivity between MMA and lithium metal makes the residual MMA monomer very likely to be the reason affecting the performance of the lithium symmetric battery (Figure S5). Previous studies have demonstrated that residual monomers are unavoidable in in situ polymerization systems [37]. In this work, thermogravimetric analysis was employed to confirm the residue of MMA monomer in the in situ polymerized electrolyte. Based on the average data from five groups of samples, the residual MMA monomer content was calculated to be approximately 4.76 wt.% (Table S1). From the test results, it can also be seen that PLE_12_ has almost no obvious mass change before and after heating, indicating that there is almost no residual THF. As mentioned above, when PLE_12_ is heated to remove THF, it possesses a certain fluidity as the temperature increases, which greatly reduces the resistance to THF volatilization and effectively solves the problem of residual solvent in the traditional solvent casting method. This result also demonstrates the excellent compatibility of PLE_12_ with the lithium metal anode, which is of profound significance for the long-term cycling stability of polymer electrolyte in practical applications. During the prolonged cycling of the lithium symmetric cell, electrochemical impedance spectroscopy was employed to measure the interfacial resistance before cycling and after a certain number of cycles. It was observed that the interfacial resistance between the electrolyte and the lithium metal electrode decreased significantly after initial cycling and subsequently stabilized (Figure 3b). This decrease and stabilization can be attributed to the formation and optimization of the solid electrolyte interphase (SEI). Initial cycling electrochemically drives the heterogeneous and porous nascent SEI to reorganize into a stable, compact, and conductive passivation layer, thereby reducing resistance and suppressing further parasitic reactions [38]. Enlarged views of the voltage profiles during the symmetric cell cycling revealed no significant distortion or fluctuation (Figure 3c–e), further confirming the formation of a stable and favorable interface between PLE12 and the lithium metal anode. The incorporation of the deep eutectic solvent enhances the ionic conductivity of PMMA while maintaining good mechanical properties (elongation at break of 287.5%). The resulting flexible contact between the electrolyte and electrodes ensures excellent interfacial contact. Furthermore, reinforcement by the PE network enables the electrolyte to effectively inhibit dendrite growth, preventing penetration.

Cycling tests of the Li|PLE_12_|Li cell were conducted at various current densities. The polarization potentials at current densities of 0.1 mA cm^−2^, 0.2 mA cm^−2^, 0.5 mA cm^−2^, and 1 mA cm^−2^ (correspond to a circular LFP cathode with a diameter of 12 mm and a mass loading of 1.2 mg charged at rates of 0.5, 1, 2.5, and 5 C.) were 0.013 V, 0.0212 V, 0.0515 V, and 0.0884 V, respectively, with stable polarization voltage maintained at each current density (Figure 3f). This effectively demonstrates that PLE_12_ can operate stably across various current densities and meet the requirements for high-rate charge–discharge applications. The critical current density of PLE_12_ was further evaluated to explore its application potential at high current density. The results showed that the critical current density of PLE_12_ was approximately 1.6 mA cm^−2^ (Figure S6). This critical current density allows PLE_12_ to cycle stably at high rates, catering to the fast-charging demands in practical applications. To further demonstrate the good compatibility of PLE_12_ with the lithium metal electrode, scanning electron microscopy was performed on pristine lithium metal and lithium metal after 400 h of operation in a Li|PLE_12_|Li cell. Observations revealed that after 400 h of cycling, no prominent sharp lithium dendrites were present on the lithium metal surface, nor was dead lithium or delamination observed (Figure 3g,h). This corroborates the effective regulation of lithium deposition achieved by combining EE with PMMA in PLE_12_. The above results indicate that PLE_12_ can effectively guide uniform lithium deposition, inhibit dendrite growth, and maintain stable interfacial characteristics under various current densities, further validating its excellent compatibility with the lithium metal anode.

### 3.4. Full-Cell Application Testing

**Figure 4:**
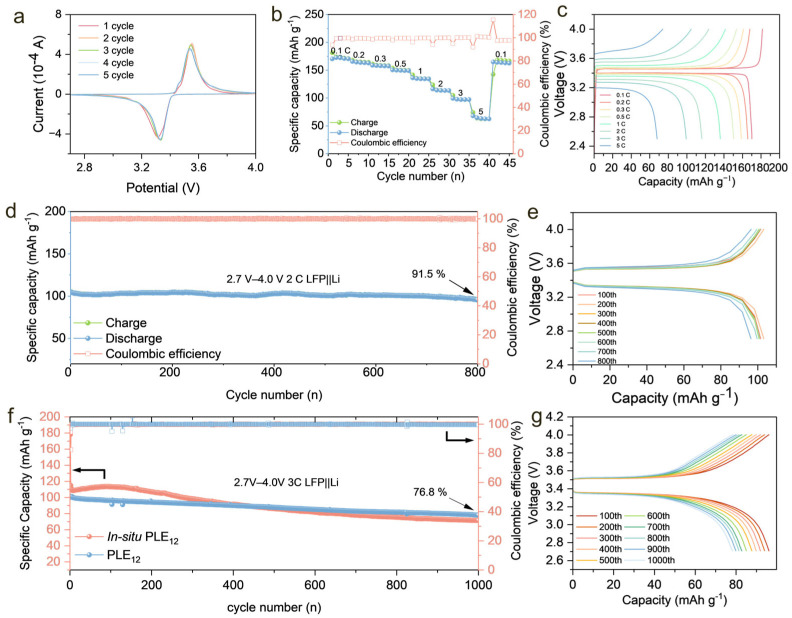
(**a**) Cyclic voltammetry curves of the LFP|PLE_12_|Li cell. (**b**) Rate capability test results of LFP|PLE_12_|Li cell cycled for five cycles at current densities of 0.1 C, 0.2 C, 0.3 C, 0.5 C, 1 C, 2 C, 3 C, and 5 C (**c**) GCD profiles of LFP|PLE_12_|Li cell cycled for five cycles at current densities of 0.1 C, 0.2 C, 0.3 C, 0.5 C, 1 C, 2 C, 3 C, and 5 C (**d**) Long-term galvanostatic charge–discharge cycling performance of the LFP|PLE_12_|Li cell at a 2 C rate. (**e**) GCD profiles of the LFP|PLE_12_|Li cell during long-term cycling at a 2 C rate. (**f**) Long-term galvanostatic charge–discharge cycling performance of LFP|PLE_12_|Li and LFP|in situ PLE_12_|Li cells at a 3 C rate. (**g**) GCD profiles of the LFP|PLE_12_|Li cell during long-term cycling at a 3 C rate.

To demonstrate the compatibility of the PLE_12_ electrolyte with the LFP electrode, cyclic voltammetry was performed on the LFP|PLE_12_|Li cell. The obtained curves show the redox peaks of the cell appearing at approximately 3.5 V/3.3 V (Figure 4a), which confirms the excellent compatibility between PLE_12_ and the LFP electrode. Rate capability tests were conducted on LFP|PLE_12_|Li cells at current densities of 0.1 C, 0.2 C, 0.3 C, 0.5 C, 1 C, 2 C, 3 C, and 5 C (1 C = 170 mAh g^−1^. The discharge specific capacities were 170.2, 165.7, 158.7, 150.5, 136.2, 116.2, 99.3, and 67.9 mAh g^−1^, respectively. Even at a high rate of 3 C, a reversible capacity of 99.3 mAh g^−1^ was maintained (Figure 4b). The GCD profiles show that the corresponding galvanostatic charge–discharge curves indicate that the LFP|PLE_12_|Li cell follows the typical Fe^2+^/Fe^3+^ redox reaction mechanism. At a rate of 0.1 C, two distinct and stable charge–discharge voltage plateaus appear at approximately 3.45 V/3.4 V, characteristic of lithium-ion deintercalation/intercalation in the LiFePO_4_ cathode [39]. As the rate increases, the voltage plateaus deviate to some extent, likely due to an increase in polarization. The polarization voltages at 0.1 C, 0.3 C, 0.5 C, 1 C, 2 C, 3 C, and 5 C were 52 mV, 70 mV, 93 mV, 146.4 mV, 240.2 mV, and 325.6 mV, respectively (Figure 4c). This mutually verifies the results obtained by cyclic voltammetry, as these low polarization voltages indicate that even at relatively high current densities, lithium ions maintain efficient transport through PLE_12_, which shows the excellent ionic conductivity performance exhibited by PLE_12_.

To further validate the application potential of PLE_12_ in practical scenarios, LFP|PLE_12_|Li cells were assembled and subjected to long-term cycling tests to verify their stability under prolonged cycling conditions. The LFP|PLE_12_|Li cell was cycled at a 2 C rate to assess the electrolyte’s performance under extended cycling demands. An initial discharge capacity of 117 mAh g^−1^ was achieved at 2 C, with a capacity retention of approximately 91.5% after 800 cycles, and the Coulombic efficiency remained above 99.8% throughout the cycling process (Figure 4d). This performance demonstrates that PLE_12_ maintains a relatively stable interface with both the cathode and anode during prolonged cycling [40], with no significant side reactions, and can meet the requirements of long-term cycling. Furthermore, observation of the long-term GCD profiles revealed that the charge–discharge plateaus remained consistently around 3.5 V/3.3 V throughout the entire cycling test, exhibiting only negligible changes in polarization voltage (Figure 4e). This also indicates that PLE_12_ maintains good conductive pathway integrity during prolonged cycling, without causing unwanted interfacial side reactions.

Given the excellent ionic conductivity performance exhibited by PLE_12_, long-term cycling tests were conducted on LFP|PLE_12_|Li cells at 3 C. The LFP|PLE_12_|Li cell delivered an initial capacity of 102 mAh g^−1^ and retained a capacity of 78 mAh g^−1^ after 1000 cycles, corresponding to a capacity retention of 76.8%, with Coulombic efficiency consistently above 99.9%. By comparison, the LFP|in situ PLE_12_|Li cell exhibited a higher initial capacity, and its stability was significantly inferior to that of LFP|PLE_12_|Li. After an initial aging period, its capacity faded more rapidly, and after 450 cycles, its capacity consistently remained lower than that of LFP|PLE_12_|Li (Figure 4f). It can also be observed from the GCD profiles of the LFP|in situ PLE12|Li cell during long-term cycling at a 3 C rate that the charge/discharge plateaus change significantly and the polarization voltage increases noticeably as the cycle number increases (Figure S7). This difference in stability may originate from the same reason as in the lithium symmetric battery, where residual MMA continuously reacts with the lithium metal anode, leading to continuous degradation of the interface between the electrolyte and lithium metal, thereby affecting the long-term cycling stability of the battery. The GCD profiles of the LFP|PLE_12_|Li cell at 3 C also show only minimal changes in polarization voltage over 1000 cycles, further illustrating the formation of a stable CEI between the PLE12 electrolyte and the cathode at high rates (Figure 4g).

### 3.5. High-Voltage Stability

**Figure 5:**
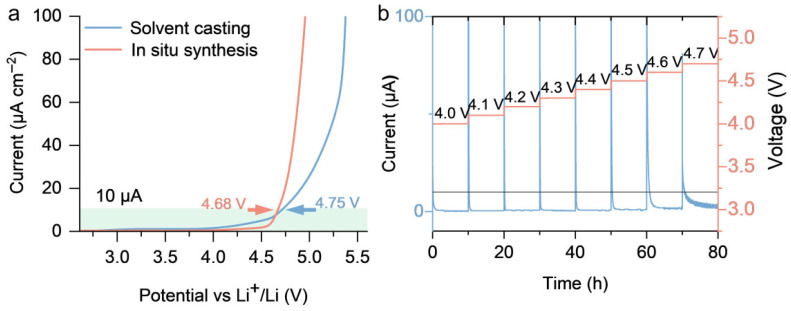
(**a**) Linear sweep voltammetry curves of SS|PLE_12_|Li and SS|in situ PLE_12_|Li cells. (**b**) Floating test results of the LCO|PLE_12_|Li cell.

To investigate the compatibility of PLE_12_ with high-voltage cathodes and evaluate its high-voltage resistance, SS|PLE_12_|Li and SS|in situ PLE_12_|Li cells were assembled for linear sweep voltammetry tests. The results show that the oxidation potential of PLE_12_ is approximately 4.75 V, higher than the 4.68 V oxidation potential of in situ PLE_12_ (potential recorded when the current density reaches 10 μA cm^−2^ (Figure 5a). An LCO|PLE_12_|Li cell was assembled for floating tests, further demonstrating the excellent oxidation resistance of PLE_12_. A consistently low current was maintained even at a voltage as high as 4.6 V (Figure 5b), showcasing outstanding oxidation resistance in conjunction with the LCO electrode.

**Figure 6:**
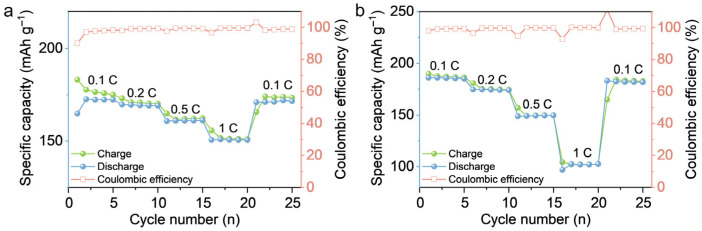
(**a**) Rate test results of LCO|PLE_12_|Li at current densities of 0.1, 0.2, 0.5, and 1 C; (**b**) Rate test results of NCM811|PLE_12_|Li at current densities of 0.1, 0.2, 0.5, and 1 C.

To verify the cycling performance of PLE_12_ in an actual high-voltage environment, LCO|PLE_12_|Li batteries were assembled and subjected to rate cycling tests at 4.5 V. From the test results, it can be seen that at current densities of 0.1, 0.2, 0.5, and 1 C, specific discharge capacities of 172.3, 169.7, 161.2, and 151.6 mAh g^−1^ were achieved, respectively, and after returning to the current density of 0.1 C, the specific discharge capacity could recover to a value close to the initial one, proving that no irreversible damage was caused to the structures of the electrodes and the electrolyte during the rate testing process, and exhibiting excellent cycling performance at 4.5 V (Figure 6a).

To verify whether PLE_12_ can maintain its high-voltage stability when combined with different high-voltage cathodes, NCM811|PLE_12_|Li batteries were assembled and subjected to rate testing at 4.5 V. From the test results, it can be seen that at current densities of 0.1, 0.2, 0.5, and 1 C, specific discharge capacities of 185.1, 173.9, 148.7, and 102 mAh g^−1^ were achieved, respectively. It is particularly noteworthy that during this rate testing process, the Coulombic efficiency remained essentially around 99.7%, which is the most powerful evidence of the high-voltage stability of PLE_12_ (Figure 6b).

## 4. Conclusions

In this work, a rigid–flexible coupled polymer electrolyte is constructed by integrating a poly(methyl methacrylate) (PMMA)/eutectic electrolyte (EE) phase into a porous polyethylene (PE) scaffold. Within this architecture, the PMMA matrix coordinates with Li^+^ through its polar carbonyl groups to promote lithium salt dissociation and form a stable ion transport network. The incorporated EE reduces the glassy rigidity of PMMA and provides continuous pathways for fast ionic conduction, while the porous PE scaffold reinforces mechanical strength and resists dendrite penetration, enabling a thin (39 μm), flexible electrolyte membrane. This structural synergy yields an ionic conductivity of 1.59 × 10^−4^ S cm^−1^ at 30 °C, a Li^+^ transference number of 0.45, and an electrochemical stability window up to 4.75 V. The electrolyte delivers stable long-term cycling in LFP|PLE_12_|Li cells, retaining 76.8% of its capacity after 1000 cycles at 3 C with Coulombic efficiency exceeding 99.9%. It also exhibits excellent high-voltage compatibility, maintaining stable performance in LCO|PLE_12_|Li and NCM811|PLE_12_|Li cells at 4.5 V. Notably, unlike in situ polymerized counterparts, this design (achieved via solution casting) eliminates residual reactive species that could otherwise compromise interfacial stability. The rigid–flexible coupled strategy thus effectively balances ionic conductivity, mechanical toughness, interfacial stability, and high-voltage tolerance, offering a promising route for high-voltage solid-state lithium-metal batteries.

## Data Availability

The original contributions presented in this study are included in the article/Supplementary Material. Further inquiries can be directed to the corresponding authors.

## Supplementary Materials

The following supporting information can be downloaded at: https://www.mdpi.com/article/10.3390/polym18080987/s1, Figure S1: (a) Electrochemical impedance spectra of PLE_1x_ (x = 1, 2, 3, 4) electrolytes (b) Electrochemical impedance spectra of PLE_1x_ (x = 0.5, 1, 2, 3, 4) electrolytes.(The thickness of the electrolyte is 39 μm, and the diameter of the stainless steel electrode is 16 mm). Figure S2: Long-term galvanostatic charge-discharge cycling performance of PLE_1x_ (x = 1, 2, 3) electrolytes at a 3 C rate. Figure S3: Polymer electrolyte samples prepared by solution-casting method and in-situ polymerization, respectively. Figure S4: Optical images of PLE_12_ without PE skeleton (a) before stretching and (b) stretched to its limit. Table S1: Thermogravimetric analysis was employed to determine the residual MMA content after in-situ polymerization. Figure S5: (a) untreated lithium metal (b) lithium metal after soaking in MMA for 2 min. Figure S6: Critical current density (CCD) test result of Li|PLE_12_|Li cell. Figure S7. GCD profiles of the LFP|in situ PLE_12_|Li cell during long-term cycling at a 3 C rate.

## Abbreviations

The following abbreviations are used in this manuscript:

PMMA	Poly(methyl methacrylate)
MMA	Methyl methacrylate
EC	Ethylene carbonate
EE	Eutectic electrolyte
PE	Polyethylene
LiTFSI	Lithium bis(trifluoromethanesulfonyl)imide
LFP	Lithium iron phosphate
NMP	N-Methyl-2-pyrrolidone
PEGDA	Poly(ethylene glycol) diacrylate
AIBN	2,2′-Azobis(2-methylpropionitrile)
LCO	Lithium cobalt oxide
PVDF	Poly(vinylidene fluoride)
SS	Stainless steel
FTIR	Fourier transform infrared spectroscopy
SEM	Scanning electron microscope
CV	Cyclic voltammetry
LSV	Linear sweep voltammetry
EIS	Electrochemical impedance spectroscopy
GCD	Galvanostatic charge–discharge
ATR	Attenuated total reflectance
