# Supplementary material for: A Polymer Electrolyte with Rigid–Flexible Coupled Architecture for High-Voltage Lithium-Metal Batteries
Source: Polymers (Basel). 2026 Apr 18;18(8):987. doi: 10.3390/polym18080987 (PMC13119575; doi:10.3390/polym18080987)
Supplement: Supplementary file 1 [file polymers-18-00987-s001.zip › polymers-4238381-supplementary.pdf]

polymers

MDPI

Supplementary Materials

A Polymer Electrolyte with Rigid–Flexible Coupled Architecture for High-Voltage Lithium-Metal Batteries

Haoru Xie [1], Zhengyin Yao [1], Zhen Liu [2], Ruiyong Chen [3,*] and Peng Zhang [1,*]

1 Key Laboratory for Polymeric Composite and Functional Materials of Ministry of Education, School of Materials Science and Engineering, Institute of Green Chemistry and Molecular Engineering, Sun Yat-sen University, Guangzhou 510275, China
2 Medical Devices Research & Testing Center, South China University of Technology, Guangzhou 510006, China
3 Department of Chemistry, University of Liverpool, Liverpool L7 3NY, UK
* Correspondence: ruiyong.chen@liverpool.ac.uk (R.C.); zhangpeng3@mail.sysu.edu.cn (P.Z.)

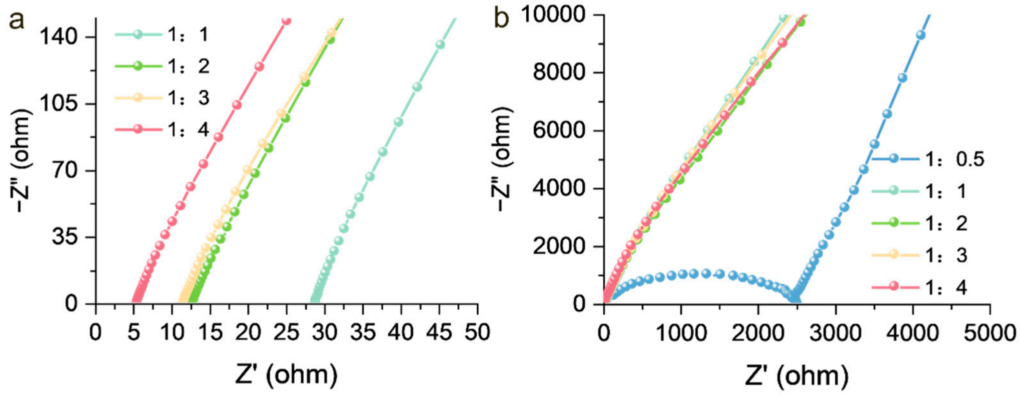

Figure S1. (a) Electrochemical impedance spectra of PLE_{1x} (x = 1, 2, 3, 4) electrolytes (b) Electrochemical impedance spectra of PLE_{1x} (x = 0.5, 1, 2, 3, 4) electrolytes.(The thickness of the electrolyte is 39 μm, and the diameter of the stainless steel electrode is 16 mm).

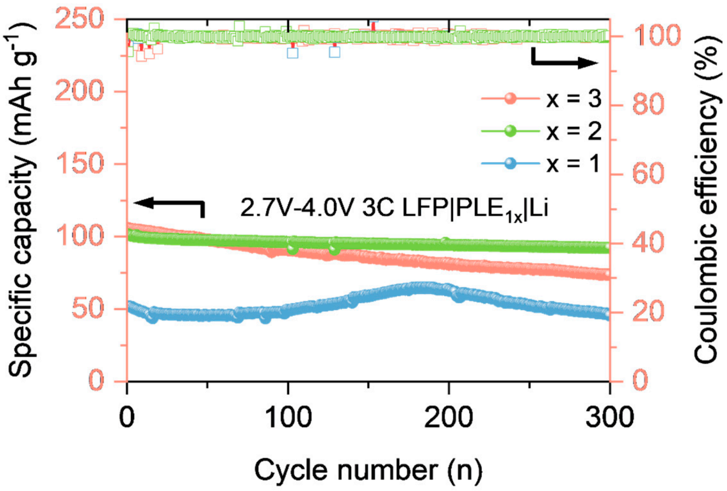

Figure S2. Long-term galvanostatic charge-discharge cycling performance of PLE_{1x} (x = 1, 2, 3) electrolytes at a 3 C rate.

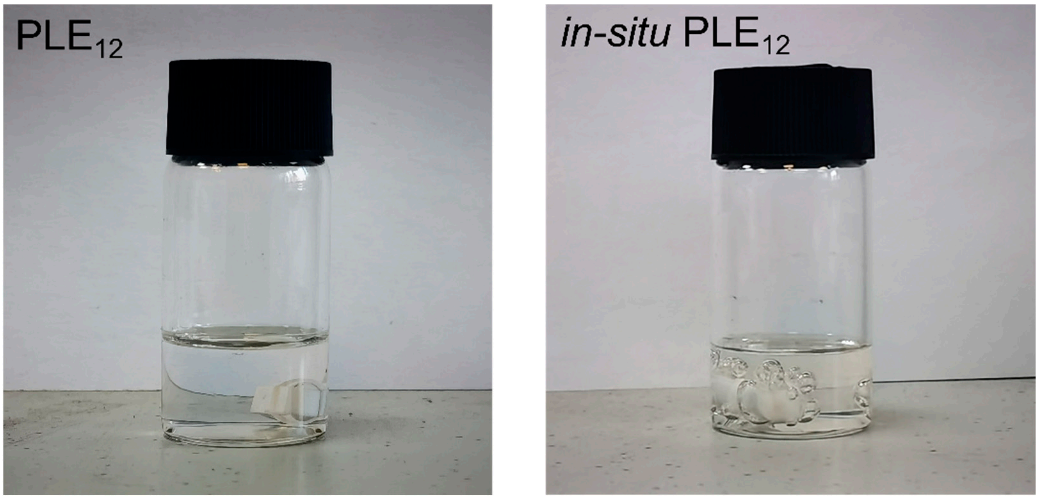

Figure S3. Polymer electrolyte samples prepared by solution-casting method and in-situ polymerization, respectively.

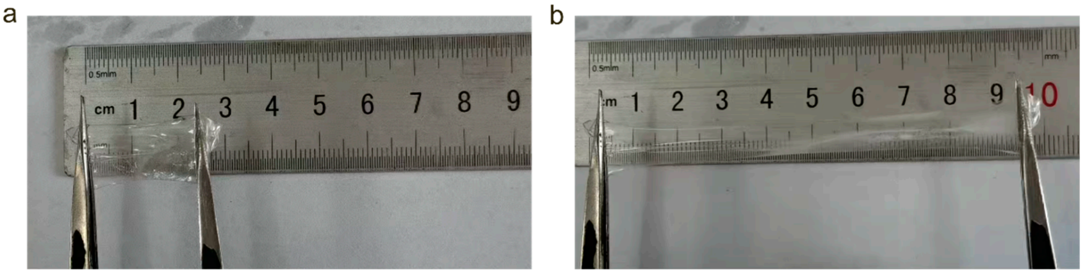

Figure S4. Optical images of PLE_{12} without PE skeleton (a) before stretching and (b) stretched to its limit.

Table S1. Thermogravimetric analysis was employed to determine the residual MMA content after in-situ polymerization.

The mass of MMA before in situ polymerization (g)	The mass of *in-situ* PLE_{12} before heating (g)	The mass of *in-situ* PLE_{12} after heating (g)	The mass of PMMA (g)	The mass of PLE_{12} before heating (g)	The mass of PLE_{12} after heating (g)
2.0028	6.0200	5.9311	1.9929	6.0104	6.0100
1.9995	6.0104	5.9095	2.0037	6.0084	6.0076
2.0102	6.0158	5.9148	2.0021	6.0106	6.0103
2.0032	6.0124	5.9233	2.0115	6.0095	6.0091
2.0211	6.0244	5.9242	2.0088	6.0133	6.0126

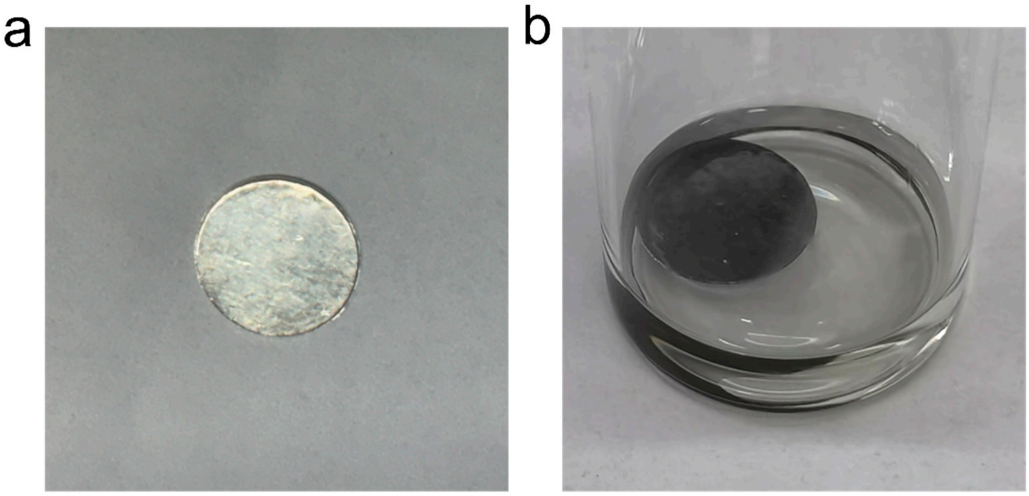

Figure S5. (a) untreated lithium metal (b) lithium metal after soaking in MMA for 2 minutes.

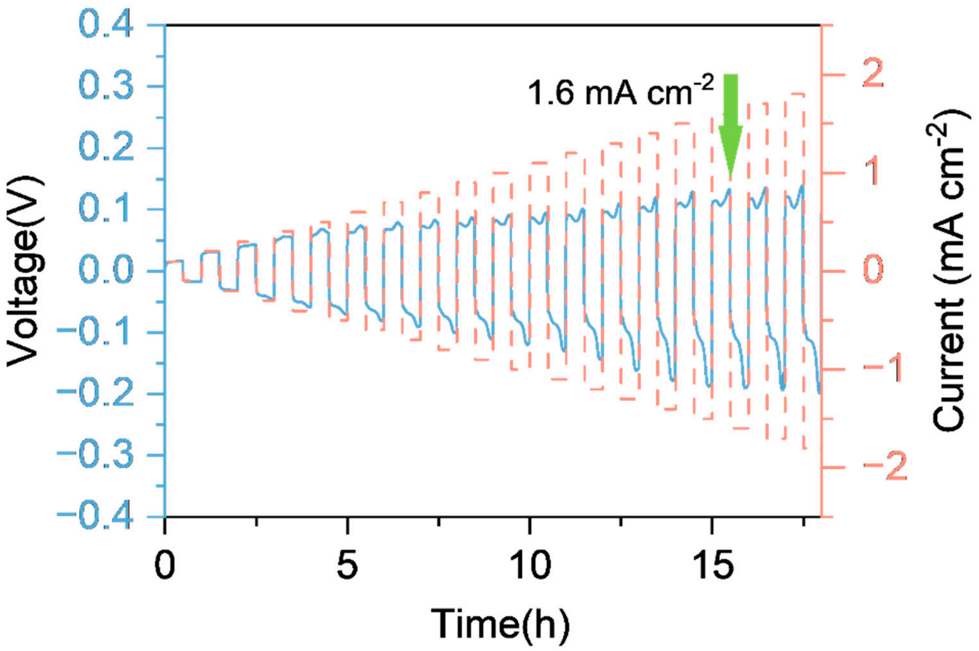

Figure S6. Critical current density (CCD) test result of Li|PLE12|Li cell.

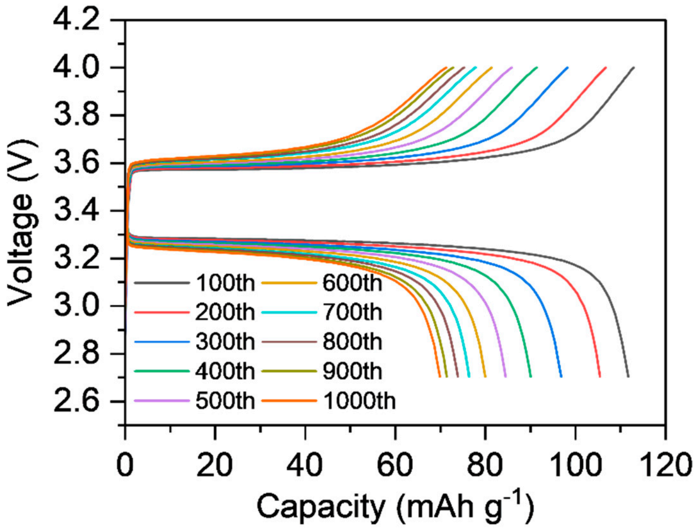

Figure S7. GCD profiles of the LFP|in situ PLE_{12}|Li cell during long-term cycling at a 3 C rate.
